# Human milk microbiome is shaped by breastfeeding practices

**DOI:** 10.3389/fmicb.2022.885588

**Published:** 2022-09-08

**Authors:** Lilian Lopez Leyva, Emmanuel Gonzalez, Noel W. Solomons, Kristine G. Koski

**Affiliations:** ^1^School of Human Nutrition, McGill University, Montreal, QC, Canada; ^2^Canadian Centre for Computational Genomics (C3G), Department of Human Genetics, McGill University, Montréal, QC, Canada; ^3^Microbiome Research Platform, McGill Interdisciplinary Initiative in Infection and Immunity (MI4), Genome Centre, McGill University, Montreal, QC, Canada; ^4^Center for Studies of Sensory Impairment, Aging and Metabolism (CeSSIAM), Guatemala City, Guatemala

**Keywords:** human milk microbiome, exclusive breastfeeding, non-exclusive breastfeeding, environmental bacteria, 16S rRNA sequencing

## Abstract

There is evidence that breastfeeding practices may impact the milk microbiota diversity and differential abundance at the genera level; however, the possibility that distinct feeding practices, such as exclusive (EBF) and non-exclusive breastfeeding (non-EBF), might alter the milk microbiome at the species level has not been explored. This cross-sectional study analyzed the milk microbiome of 64 *Mam-*Mayan indigenous mothers from San Juan Ostuncalco in Guatemala. Two breastfeeding practices [exclusive (EBF) vs non-exclusive (non-EBF)] were analyzed at two stages of lactation [early (5–46 days post-partum) vs late (109–184 days post-partum)]. EBF was defined as offering only human milk and non-EBF was defined as feeding the infant herbal teas (*agüitas*) and/or complementary foods while continuing to breastfeed. Results identified four clusters with distinct microbial communities that segregated bacterial species by both breastfeeding practices and stage of lactation. Comparison among these clusters identified several notable patterns. First, during EBF, the microbiome differed by stage of lactation where there was a shift in differential abundance from Actinobacteria and Firmicutes in early to Bacteroidetes and Proteobacteria species in late lactation. Second, a similar comparison between non-EBF mothers by stage of lactation also identified a higher differential abundance of Actinobacteria and Firmicutes species in early lactation, but only Proteobacteria and not Bacteroidetes in late lactation, indicating a further shift in the milk microbial ecosystem with fewer oral bacteria present in late lactation. Third, comparisons between EBF and non-EBF mothers at both early and late lactation showed that mothers who exclusively breastfed had more differentially abundant species in early (11 vs 1) and late (13 vs 2) lactation. Fourth, EBF at early and late lactation had more commensal and lactic acid bacteria, including *Lactobacillus gasseri, Granulicatella elegans, Streptococcus mitis*, and *Streptococcus parasanguinis*, compared to those who did not exclusively breastfeed. Collectively, these results show that EBF has more differentially abundant bacteria, including commensal and lactic acid bacteria, and that the addition of *agüitas* (herbal teas) and/or complementary foods modify the milk microbiome composition by reducing the oral bacteria and introducing more environmentally sourced bacteria to the ecosystem.

## Introduction

Research on human milk microbiota has increased recently due to growing interest in understanding factors shaping the milk microbiome (Fitzstevens et al., 2017; Zimmermann and Curtis, 2020; Lopez Leyva et al., 2021a). Several studies in developed countries, where exclusive breastfeeding to 6 months is limited (UNICEF, 2019), have analyzed the human milk microbiota of breastfed and formula-fed infants (Bokulich et al., 2016; Martin et al., 2016; Carvalho-Ramos et al., 2018), noting that exclusive breastfeeding for 2 months was associated with healthier gut bacterial communities (Ho et al., 2018). In contrast, developing countries often report higher rates of exclusive breastfeeding for up to 6 months than developed countries (UNICEF, 2019). In fact, in Guatemala, exclusive breastfeeding rates exceed 53% for the first 5 months, and in rural communities, the prevalence increases to 61% compared to 41% worldwide (UNICEF, 2019). However, few studies have investigated breastfeeding practices in developing countries (Lopez Leyva et al., 2021a). In LMIC (low- and middle-income countries), cultural practices in early feeding may include ritual fluids. This is a common practice in Mexico (Deming et al., 2015; Rodríguez-Ramírez et al., 2016; Afeiche et al., 2018), Bangladesh, Brazil, India, Nepal, Pakistan, Peru, South Africa, and Tanzania (Patil et al., 2015). These ritual fluids or herbal teas are usually culturally ‘prescribed’ to maintain infant health (Wren et al., 2015) and to treat gastrointestinal infections, colic, stomach pain, constipation, sore throat, or fever (Doak et al., 2013), and for irritability and crying (Chomat et al., 2015).

The possibility that these ritual fluids and the introduction of complementary food might further impact the human milk microbiome has not been widely considered. Interestingly, there is emerging evidence to suggest that environmental bacteria in soil and water may function as a superorganism that can replenish human microbial communities as inoculants and provide beneficial microorganisms which could positively impact human health (Blum et al., 2019). Some of the benefits assigned to the interaction of bacteria present in the environment and the human microbiome include less propensity for allergies (Hanski et al., 2012), suppression of soil-borne pathogens, exposure to immunoregulation-inducing soil microorganisms, immune tolerance, and an increase in microbial diversity (Wall et al., 2015). Soil or water genera identified in human milk include *Acinetobacter* (Kumar et al., 2016; Sakwinska et al., 2016; Urbaniak et al., 2016; Drell et al., 2017; Patel et al., 2017), Bradyrhizobiaceae (Hunt et al., 2011), *Novosphingobium* (Jiménez et al., 2015), *Pseudomonas* (Hunt et al., 2011; Jiménez et al., 2015; Sakwinska et al., 2016; Pannaraj et al., 2017; Patel et al., 2017; Moossavi et al., 2019), *Ralstonia* (Hunt et al., 2011; Kumar et al., 2016; Vaidya et al., 2017; Williams et al., 2017; Moossavi et al., 2019), *Sphingobium* (Jiménez et al., 2015; Mueller et al., 2015), *Sphingomonas* (Hunt et al., 2011; Urbaniak et al., 2014; Jiménez et al., 2015; Davé et al., 2016; Kumar et al., 2016; Li et al., 2017; Ding et al., 2019; Hermansson et al., 2019), and *Stenotrophomonas* (Urbaniak et al., 2014; Davé et al., 2016). Although still debated (Salter et al., 2014; Jiménez et al., 2015; Sakwinska et al., 2016; Douglas et al., 2020), some of these environmental genera, such as Bradyrhizobiaceae, *Pseudomonas, Sphingomonas*, and *Ralstonia*, have been identified as part of the “core” human milk microbiome (Hunt et al., 2011; Jiménez et al., 2015). It has been suggested that human milk acquired these environmental bacteria through a maternal diet based on legumes (Drago et al., 2017), proximity to soil environments (Blum et al., 2019), and/or agrarian lifestyles (Yatsunenko et al., 2012; Clemente et al., 2015; Meehan et al., 2018).

Recently, we conducted a study in the *Mam*-Mayan indigenous community in Guatemala to identify maternal factors involved in modifying the human milk microbiome at both the genera (Lopez Leyva et al., 2021b) and species levels (Gonzalez et al., 2021) and identified distinct clustering of microbial communities associated with both stages of lactation and breastfeeding practices. The study conducted at the species level reported a shift from *Staphylococcus* and *Streptococcus* species in early lactation to *Sphingobium* and *Pseudomonas* species in late lactation (Gonzalez et al., 2021). However, in this later study, breastfeeding practices were not considered.

To date, no studies have investigated the relationships between the stage of lactation and breastfeeding practices in the shaping of the human milk microbiome at the species level. This is important because reporting at the species level provides more information about the functionality and facilitates biological interpretation by having an improved resolution of the data (Lopez Leyva et al., 2021a). The purpose of this study was to explore how two breastfeeding practices [exclusive (EBF) vs non-exclusive breastfeeding (non-EBF)] at two stages of lactation [early (5–46 days) vs late (109–184 days)] might modify the human milk microbiome. Our specific objectives were (1) to characterize the milk microbiome of EBF in early and late lactation in mothers living in eight rural *Mam*-speaking indigenous communities in Guatemala and (2) to compare shifts between mothers who exclusively breastfed vs those who did not exclusively breastfeed (EBF and non-EBF) at each stage of lactation.

## Materials and methods

### Study site and participants

This cross-sectional study was part of a collaboration between McGill University and the Center for Studies of Sensory Impairment, Aging, and Metabolism (CeSSIAM) in the Republic of Guatemala. Field studies were conducted from June 2012 through January 2013 in eight rural *Mam*-speaking communities of the San Juan Ostuncalco region in Guatemala (Chomat et al., 2015). The inclusion criteria were indigenous lactating women with infants at (1) early stage of lactation (5–46 days) or (2) late stage of lactation (109–184 days) and who had a vaginal delivery. The exclusion criteria were: (1) mothers with colostrum (milk < 4 days post-partum) and (2) mothers treated with antibiotics during the post-partum period. Ethical approval was obtained from the Institutional Review Boards of both institutions, and permission was obtained from community leaders and the local authorities of the Ministry of Health. All mothers provided written informed consent for participation in the study.

### Study design

Two breastfeeding practices [exclusive (EBF) vs non-exclusive (non-EBF)] were analyzed at two stages of lactation [early (5–46 days post-partum) vs late (109–184 days post-partum)]. Exclusive breastfeeding (EBF) was defined as providing only human milk to the infant and non-exclusive breastfeeding (non-EBF) at the early stage was defined as providing water or *agüitas* in addition to human milk. *Agüitas* are ritual fluids; the infusions more commonly used are boiled water, sugar water, chamomile tea, corn paste water, anise water, orange leaf water, mint water, or sage water. Non-EBF at the late stage was defined as providing *agüitas* and/or complementary foods to the infant while they continued to breastfeed. From the sample of 64 women, the four groups created were early EBF (*n* = 15), early non-EBF (*n* = 14), late EBF (*n* = 18), and late non-EBF (*n* = 17).

The classification of the type of breastfeeding practice into groups was done based on a structured, in-depth questionnaire administered to mothers about independent factors (maternal, infant, and cultural practices and beliefs) that may influence breastfeeding initiation, exclusivity, and frequency (Wren et al., 2015). Trained local health care workers administered the questionnaire orally in either Spanish or *Mam* to participants during a 30- to 40-min interview (Wren et al., 2015). Feeding patterns were defined as EBF or non-EBF based on cumulative recall since birth. Mothers were asked if they ever fed their infant *agüitas* since birth (yes/no). If yes, the timing of the *agüitas* initiation was queried (hours, days, and weeks post-partum). Likewise, mothers were asked to identify from a list the type of *agüitas* given to the infant and provide an open-ended reason (Wren et al., 2015).

### Human milk sample collection

Milk samples from early lactation (5–46 days post-partum) and late lactation (109–184 days post-partum) were collected during the period from 2012 to 2013. These ranges were chosen to be consistent with our previous studies that measured infant growth (Li et al., 2016, 2019; Wren-Atilola et al., 2018). Prior to collection, the nipple and areola of the breast were cleaned with 70% ethyl alcohol. Human milk samples were collected during a 3-h time window in the morning from the breast not recently used for breastfeeding via full manual expression by a trained midwife, who used hand sanitizer before and after collection. This is important since other studies have shown differences in milk microbiome diversity with the use of breast pumps (Moossavi et al., 2019). Milk was collected into 60 ml plastic vials and immediately stored on ice. Samples were partitioned into 15 ml tubes at the field laboratory (−30°C) prior to transfer on dry ice to McGill University where they were stored at −80°C, which is known to preserve the milk microbiome integrity (Lyons et al., 2021), until DNA extraction for microbiome analysis was performed in 2018.

### 16S rRNA gene amplification, sequencing, bioinformatics, and statistical analysis

Methodology (sequencing and microbiome characterization) is as described in Gonzalez et al., 2021. Briefly, DNA extraction was done using 1 ml of milk with DNeasy Blood and Tissue mini kit from Qiagen according to the manufacturer’s protocol. For PCR, a region of ∼526 bp in the 16S rRNA gene, covering the V1–V3 region, was amplified with the universal eubacterial primers 27F: AGAGTTTGATCCTGGCTCAG and 533R: TTACCGCGGCTGCTGGCAC (Cabrera-Rubio et al., 2012). The subsequent 16S rRNA gene sequencing was performed using the Illumina MiSeq platform at McGill University and Génome Quebec Innovation Centre. Amplicons were assembled from 300 paired-end reads. Reagent controls were below the detection limit used by Génome Quebec Innovation Centre for quality assurance. Microbial data processing was performed using ANCHOR, a 16S rRNA gene amplicon pipeline for microbial analysis (Gonzalez et al., 2019). Briefly, Mothur (Schloss et al., 2009) was used to align and dereplicate sequences before high-count OTU selection at a count threshold of 36 across all samples. NCBI 16S rRNA RefSeq, NCBI non-redundant nucleotide, SILVA, and the Ribosomal Database Project (RDP) databases were used to annotate OTUs using BLASTn with criteria of > 99% for identity and coverage. When a BLASTn return had 100% identity and coverage hits across multiple databases, priority was given to NCBI 16S rRNA RefSeq due to the high standard of curation. Low counting amplicons (<36 counts) were binned to high-count OTUs at a lower threshold of >98% identity/coverage with multiple, equally good (highest identity/coverage); all the annotation was retained and reported. However, all annotation, and in particular species calls, should be considered putative even when sharing 100% sequence identity to a single species due to database errors. Bacteria with errors in the data repositories which qualified as unknown bacteria were identified as “DBinconsistency_MS” for multiple species or “DBinconsistency_MG” for multiple genera. Contamination was controlled at all stages of this analysis. This included the use of aseptic sampling protocols monitored by trained specialists as well as PCR blanks performed at Génome Quebec in Canada (no samples were sequenced if any bands were visible in negative controls). Contamination control continued at the bioinformatics stage where the Canadian Centre in Computational Genomics of McGill University carried out contemporary investigation and control via sample pre-processing including ordination analysis, control for sparsity and prevalence, and identifying putative contamination (decontam, R package; Supplementary File 1). Finally, the biostatistics analysis involved a differential abundance test (DESeq2) which contained a Cook’s distance analysis step prior to the test.

Alpha diversity of human milk samples was measured using Chao 1, Shannon, and Observed indices within the Phyloseq R package (McMurdie and Holmes, 2013). Significant differences between alpha-diversity indices were estimated using a *t*-test (for normally distributed values) or a non-parametric method (Mann–Whitney test). *P*-values were corrected for multiple comparisons using the Benjamini-Hochberg procedure. Beta diversity was assessed using the constrained correspondence analysis (CCA) ordination method. Dispersion ellipses were drawn using the veganCovEllipse function from the Vegan package in R (Oksanen et al., 2020). The significance of the different constraints in the CCA analysis was evaluated using an ANOVA-like permutation test (vegan package).

To characterize differentially abundant taxonomic units between groups of samples, parametric models developed in transcriptomics perform well when applied to microbiome biomarker data (uneven library sizes, sparsity, and sample representativity) (Gonzalez et al., 2015; Jonsson et al., 2016; Weiss et al., 2017; Minerbi et al., 2019; Brereton et al., 2020). DESeq2 procedure (Love et al., 2014) was used to calculate differentially abundant taxonomic units. Taxonomic units tested with a false discovery rate (FDR or the expected proportion of false-positive findings) <0.1 were considered significant (Anders et al., 2013; Love et al., 2014, 2016; Gonzalez et al., 2019).

## Results

### Maternal characteristics and breastfeeding practices

Population characteristics are summarized in Table 1. The participating mothers were categorized into four groups depending on the stage of lactation (early or late) and breastfeeding practices (EBF or non-EBF). In early lactation, 51.7% of infants were EBF and 48.3% were non-EBF (added *agüitas*), whereas in late lactation, 51.4% were EBF and 48.6% were non-EBF (22.9% provided *agüitas* and human milk and 25.7% provided *agüitas*, complementary foods, and human milk). With regard to other breastfeeding practices, 59% breastfed within the first hour, and the average frequency of breastfeeding was 11 ± 3.5 feeds per day.

**TABLE 1:** Maternal characteristics and breastfeeding practices.

Maternal factors	Population characteristics	Early stage (5–46 days)		Late stage (109–184 days)	
		Exclusive	Non-Exclusive	Exclusive	Non-Exclusive
		EBF	Non-EBF	EBF	Non-EBF
*N*/%	64	15 (51.7)	14 (48.3)	18 (51.4)	17 (48.6)
Age, y	23.6 ± 5.9	24.3 ± 5.9	22.3 ± 4.9	23.8 ± 4	24 ± 8.1
BMI, kg/m^2^	23.8 ± 3.3	22.8 ± 2.6	24.5 ± 3.1	23.8 ± 3.6	24.1 ± 3.8
Normal, kg/m^2^	22.2 ± 2	21.5 ± 2	22.9 ± 2	22.1 ± 2	22.5 ± 1.7
Overweight, kg/m^2^	28 ± 3	26.2 ± 2	28.5 ± 1	28.1 ± 2.9	29.3 ± 4.2
Normal, %	73.4	73.3	71.4	72.1	76.5
Overweight, %	26.6	26.7	28.6	27.8	23.5
**Parity, %**					
Primiparous	44.4	33.3	50	29.4	64.7
Multiparous	55.6	66.7	50	70.6	35.3
**Education, %**					
No	77.4	86.7	85.7	76.5	62.5
Primary education or higher	22.6	13.3	14.3	23.5	37.5
**Breastfeeding practices**					
Breastfeeding in first hour, %	59	64.3	42.9	70.6	56.25
Breastfeeding frequency, times/day	11.4 ± 3.5	12.3 ± 3.8	11.3 ± 3.3	10.4 ± 3.2	11.8 ± 3.7
**Infant first food, %**					
Breast	88.7	100	78.6	100	75
*Agüitas*	11.3	0	21.4	0	25

### Bacterial characteristics of the human milk

A total of 503 OTUs were assembled and captured 77,827 sequence reads across all 64 human milk samples (Supplementary File 2). These could be annotated as 287 OTUs, 134 genera, and 76 family or higher taxa, as well as 109 which could not be recognized as 99% similar (in both identity and coverage) to any known taxa and were labeled as unknown. Of the 287 OTUs annotated as putative species, the average BLASTn return identity was 99.8% including 173 perfect hits (100% identity). Main factors were projected onto an unconstrained ordination diagram (NMDS), and each variable regression was independently tested by Monte Carlo permutation (envfit function from R package Vegan). The goodness of fit obtained is described in Supplementary File 3.

**FIGURE 1:**
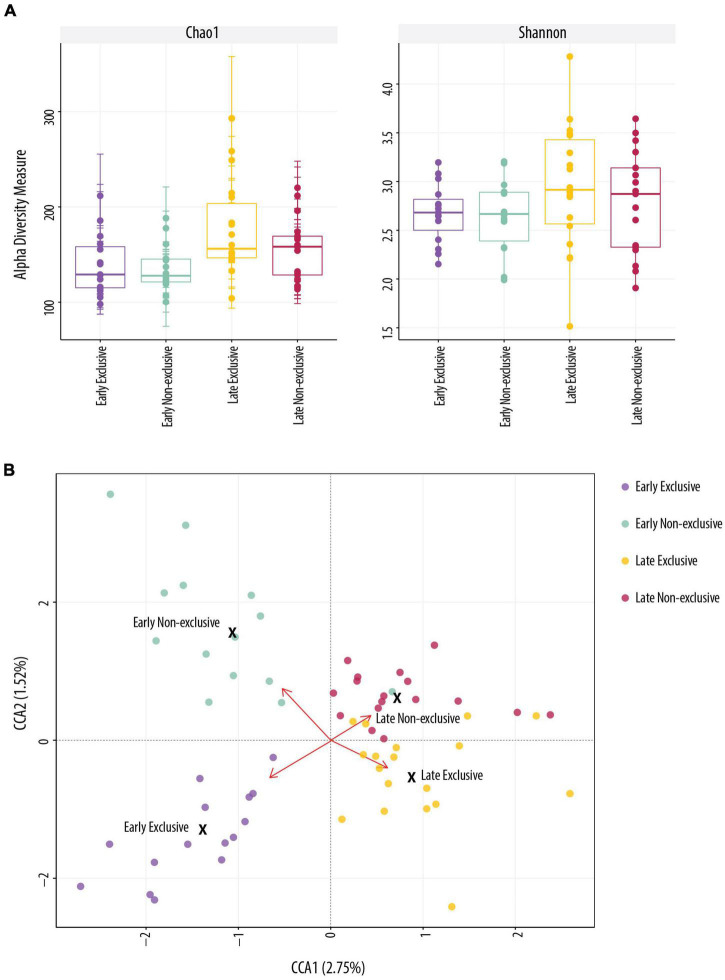
Alpha and beta diversity. **(A)** Alpha diversity indices were not significantly different in Shannon (*t*-test, *p* > 0.05) index across the four groups. Chao 1 was significant (*t*-test, *p* < 0.05) between early EBF (*n* = 15) and late EBF (*n* = 18). **(B)** Beta-diversity analysis using constrained correspondence analysis (CCA) ordination representation for each breastfeeding practice (Exclusive and Non-Exclusive) at early and late lactation stages (=0.021).

Figure 1 shows the estimation of alpha diversity (A) and beta diversity (B). The indices Chao 1, Shannon, and Observed used to estimate alpha diversity identified differences between early and late lactation for EBF and non-EBF mothers. Differences in alpha diversity using Chao 1 were significant between early non-EBF and late EBF (FDR = 0.01) and between early EBF and late EBF (FDR = 0.04); using Observed, there were significant differences between early EBF and late EBF (FDR = 0.002), early non-EBF and late EBF (FDR = 0.005), and late EBF and late non-EBF (FDR = 0.08; Supplementary File 4). In beta-diversity analysis, CCA ordination significantly segregated (*p* = 0.021) the four groups: early EBF, early non-EBF, late EBF, and late non-EBF.

### Comparison of milk microbiome in exclusive breastfeeding during early (5–46 days) and late lactation (109–184 days)

**FIGURE 2:**
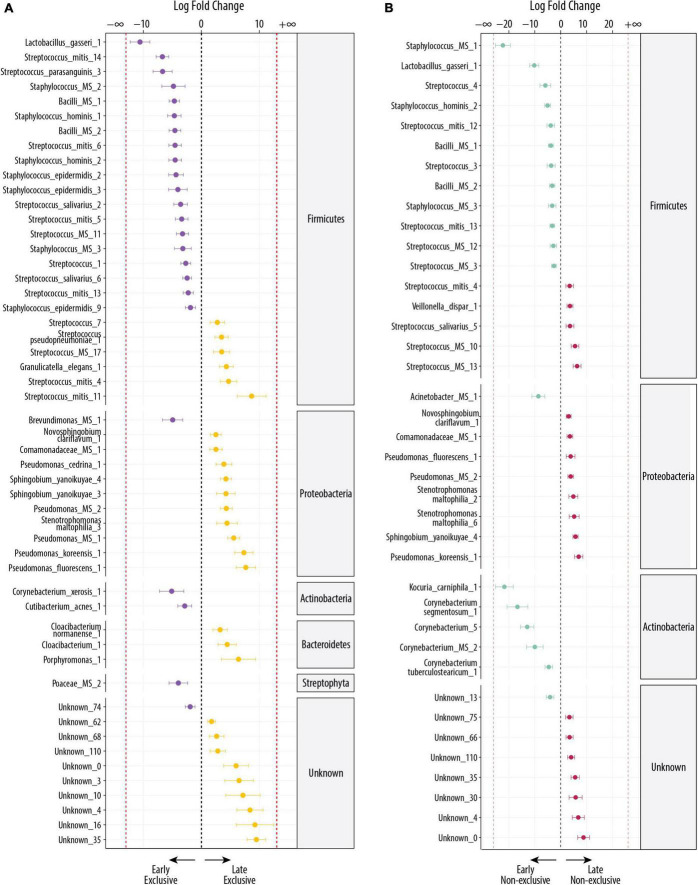
Differentially abundant bacteria associated with the lactation stage. Significantly different OTUs between groups were estimated using DESeq (FDR < 0.1). Species are grouped by phylum and ordered by logFC in each group. The dashed red line indicates “infinite” log fold change, where an OTU had detectable counts in samples from only a single group. **(A)** Differentially abundant OTUs between the early EBF (*n* = 15) and late EBF (*n* = 18) groups. Fifty-two OTUs were differentially abundant, of which 24 were more abundant at early EBF and 28 at late EBF. **(B)** Differentially abundant OTUs between the early non-EBF (*n* = 14) and late non-EBF (*n* = 17) groups. Thirty-nine OTUs were differentially abundant, of which 20 were more abundant at early non-EBF and 19 at late non-EBF.

The differential abundance (DA) analysis between EBF at early and late stage identified 52 significant differentially abundant OTUs (FDR < 0.1), from which 24 OTUs were more abundant in the early stage and 28 OTUs in the late stage (Figure 2A). OTUs from Actinobacteria and Firmicutes were more abundant at the early stage, whereas at the late stage, Bacteroidetes and Proteobacteria were more abundant. The OTUs with the highest fold change (log2 FC ≤ −5) at early stage were the commensal and lactic acid bacteria: *Lactobacillus gasseri_1* [false discovery rate (FDR) = 2.16 × 10^–8^; log2 FC = −11], the oral bacteria *Streptococcus mitis_14* (FDR = 2.16 × 10^–8^; log2 FC = −7), *Streptococcus parasanguinis_3* (FDR = 4.5 × 10^–4^; log2 FC = −7), and *Corynebacterium xerosis_1*, bacteria commonly found on the skin (FDR = 0.05; log2 FC = −5).

At late stage, the OTUs with the highest log2 FC (log2 FC ≥ 5), excluding the ones labeled as unknown were *Streptococcus mitis_11* (FDR = 0.003; log2 FC = 9), a species commonly found in the oral cavity, followed by OTUs commonly found in the environment, including *Pseudomonas fluorescens_1* (FDR = 6 × 10^–5^; log2 FC = 8), *Pseudomonas koreensis_1* (FDR = 4.35 × 10^–5^; log2 FC = 7), *Porphyromonas_1* (FDR = 0.03; log2 FC = 6), and *Pseudomonas MS_1* (FDR = 1.82 × 10^–6^; log2 FC = 6).

### Comparison of milk microbiome in non-exclusive breastfeeding during early (5–46 days) and late lactation (109–184 days)

The DA analysis between non-EBF at the early and late stage identified 39 significant differential abundant OTUs (FDR < 0.1), from which 20 OTUs were more abundant in the early stage and 19 in the late stage (Figure 2B). The shift observed in EBF groups across early and late stages repeats in the non-EBF groups, where OTUs from Actinobacteria and Firmicutes dominated at the early stage and Proteobacteria at the late stage. In contrast to late EBF, OTUs from Bacteroidetes were not differentially abundant in non-EBF groups. The OTUs with the highest log2 FC (log2 FC ≤ −5) in early non-EBF were a mix of oral and environmental bacteria, but there was still a high presence of bacteria commonly found in oral cavity and human tissues: *Staphylococcus MS_1*, which shows 100% match with *Staphylococcus haemolyticus*, a species commonly found in human tissues (FDR = 4.9 × 10^–13^; log2 FC = −22), *Kocuria carniphila_1* (FDR = 5.38 × 10^–9^; log2 FC = −22), *Corynebacterium segmentosum_1* (FDR = 2.84 × 10^–4^; log2 FC = −17), the OTU *Corynebacterium_5* (FDR = 4.61 × 10^–6^; log2 FC = −13), *Lactobacillus gasseri_1* (FDR = 1.01 × 10^–7^; log2 FC = −10), *Corynebacterium MS_2*, which could be *C. jeikeium, C. amycolatum*, or *C. xerosis* (FDR = 0.01; log2 FC = −10), *Acinetobacter MS_1* which could be *A. guillouiae* or *A. lwoffii* (FDR = 0.004 × 10^–5^; log2 FC = −9), the OTU *Streptococcus_4* (FDR = 0.02; log2 FC = −6), and *Staphylococcus hominis_2* (FDR = 6.88 × 10^–5^; log2 FC = −5).

The OTUs with the highest log2 FC (log2 FC ≥ 5) in late non-EBF, excluding the OTUs labeled as unknown, were predominantly environmental bacteria: *Pseudomonas koreensis_1* (FDR = 8.17 × 10^–4^; log2 FC = 7), *Streptococcus MS_13* (FDR = 2.23 × 10^–4^; log2 FC = 6), *Sphingobium yanoikuyae_4* (FDR = 1.12 × 10^–7^; log2 FC = 6), *Streptococcus MS_10* (FDR = 0.002; log2 FC = 5), and *Stenotrophomona maltophilia_6* (FDR = 0.03; log2 FC = 5).

### Comparison of milk microbiome in exclusive breastfeeding vs non-exclusive breastfeeding during early lactation (5–46 days)

**FIGURE 3:**
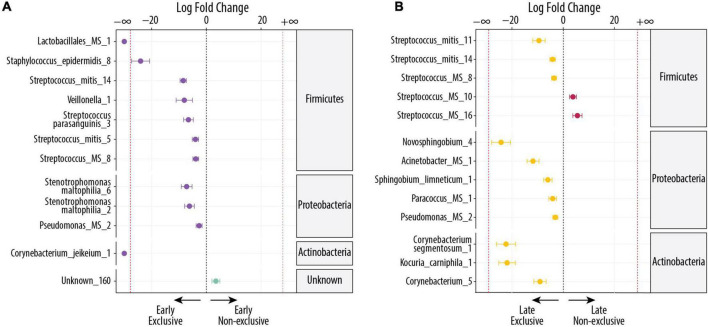
Differentially abundant bacteria associated with EBF and non-EBF. Significantly different OTUs between groups were estimated using DESeq (FDR < 0.1). Species are grouped by phylum and ordered by logFC in each group. The dashed red line indicates “infinite” fold change, where an OTU had detectable counts in samples from only a single group. **(A)** Differentially abundant OTUs between the early EBF (*n* = 15) and early non-EBF (*n* = 14) groups. Twelve OTUs were differentially abundant, of which 20 were more abundant in early EBF and 1 in early non-EBF. **(B)** Differentially abundant OTUs between the late EBF (*n* = 18) and late non-EBF (*n* = 17) groups. Thirteen OTUs were differentially abundant, of which 11 were more abundant in late EBF and 2 in late non-EBF.

During early lactation, EBF mothers had more DA OTUs than non-EBF mothers. Figure 3A shows the 12 DA in EBF vs non-EBF mothers (FDR < 0.1), where 11 had higher relative abundance in early EBF and 1 had higher relative abundance in early non-EBF. Early EBF was the only group where two OTUs were identified as unique to the group. These were *Corynebacterium jeikeium_1* (FDR = 7.08 × 10^–4^; log2 FC = −16) and *Lactobacillales MS_1* (FDR = 0.02; log2 FC = −7). The first one is a species commonly found in the environment, and the second one could be *Abiotrophia paraadiacens* or *Granulicatella adiacens.* The rest of the OTUs more abundant in early EBF were a mix of oral, human tissue, and environmental bacteria: *Staphylococcus epidermis_8* (FDR = 2.04 × 10^–11^; log2 FC = −24), *Streptococcus mitis_14* (FDR = 2.04 × 10^–11^; log2 FC = −8), *Veillonella_1* (FDR = 0.05; log2 FC = −8), *Stenotrophomonas maltophilia_6* (FDR = 0.002; log2 FC = −7), *Streptococcus parasanguinis_3* (FDR = 0.002; log2 FC = −7), *Stenotrophomonas maltophilia_2* (FDR = 0.004; log2 FC = −6), *Streptococcus mitis_5* (FDR = 0.005; log2 FC = −4), *Streptococcus_MS_8* (FDR = 0.003; log2 FC = −4), and *Pseudomonas_MS_2* (FDR = 0.1; log2 FC = −3), The only OTU DA in early non-EBF was unknown_160 (FDR = 0.1; log2 FC = 4).

### Comparison of milk microbiome in exclusive breastfeeding vs non-exclusive breastfeeding during late lactation (109–184 days)

During late lactation, the differences in DA OTUs between EBF and non-EBF mothers were similar to early lactation where EBF had more OTUs than non-EBF (Figure 3B). Of the 13 OTUs that were DA (FDR < 0.1), 11 OTUs belonged to late EBF and 2 OTUs to late non-EBF. In relation to the source, six of the 13 OTUs were identified as commonly found in the environment, and the rest (seven) were identified as commensal and commonly found in the oral cavity or human tissues. The OTUs in EBF with the highest log2 FC (log2 FC ≤ −5) were as follows: *Novosphingobium_4* (FDR = 2.78 × 10^–9^; log2 FC = −24), *Corynebacterium segmentosum_1* (FDR = 9.96 × 10^–8^; log2 FC = −22), *Kocuria carniphila_1* (FDR = 9.11 × 10^–10^; log2 FC = −22), *Acinetobacter_MS_1* (FDR = 1.97 × 10^–5^; log2 FC = −12), *Streptococcus mitis_11* (FDR = 0.002; log2 FC = −9), *Corynebacterium_5* (FDR = 0.004; log2 FC = −9), and *Sphingobium limneticum_1* (FDR = 0.006; log2 FC = −6).

The only more abundant OTUs in late non-EBF were *Streptococcus MS_16* (FDR = 0.03 × 10^–4^; log2 FC = 6), which could be *S. mitis or S. pneumoniae* and *Streptococcus MS_10* (FDR = 0.08; log2 FC = 4), which could be *S. mitis, S. oralis, S. pneumoniae*, or *S. pseudopneumoniae*; all were OTUs commonly found in the oral cavity or human tissues (Figure 3B).

## Discussion

To our knowledge, this is the first study to analyze the impact of exclusive and non-exclusive breastfeeding on the human milk microbiome at the OTU level and to determine whether different breastfeeding practices further modified the milk microbiome during early and late lactation. Additional novel findings reported in this study include the following. When comparing the lactation stages in EBF, early lactation was associated with a higher relative abundance of commensal, oral, and lactic acid bacteria, whereas at late lactation, we observed several species commonly found in the environment. In non-EBF at early lactation, although there was a mix of bacteria of the oral cavity, bacteria of human tissues origin, and environmental bacteria, bacteria commonly found in the oral cavity and human tissues showed a higher presence than the environmental bacteria. Finally, milk of non-EBF at late lactation had predominantly environmental bacteria.

### Dominant bacteria

*Streptococcus* emerged as a dominant genus among all breastfeeding practices, but the OTUs differed by stage of lactation and by breastfeeding practice. *S. mitis* was observed in all breastfeeding practices, *S. salivarius* in early EBF and late non-EBF, and *S. parasanguinis* in early and late EBF. *Streptococcus* has been considered part of the “core” microbiota in human milk (Hunt et al., 2011; Cabrera-Rubio et al., 2012; Lackey et al., 2019; Moossavi et al., 2019), and in fact, the few studies that have reported results at the species level have also reported *Streptococcus salivarius* and *Streptococcus mitis* as part of the dominant species in human milk (Heikkilä and Saris, 2003; González et al., 2013; Jost et al., 2013). They are known prevalent species in the oral cavity of infants (Könönen, 2000). Of importance, *Streptococcus salivarius* is capable of suppressing the growth of pathogenic bacteria, such as *Staphylococcus aureus*, and it has also been reported as a putative probiotic (Damaceno et al., 2017).

In addition to *Streptococcus* (Hunt et al., 2011; Boix-Amorós et al., 2016), other dominant bacteria observed in the present study aligned with previous findings at the genera level. These included the presence of bacteria from the genera *Pseudomonas* (Hunt et al., 2011; Jeurink et al., 2013; Boix-Amorós et al., 2016), *Sphingobium*, and *Novosphingobium* (Jeurink et al., 2013). However, there is still a debate as to whether *Novosphingobium* and *Pseudomonas* are part of the “core” microbiota of human milk or contaminants (Salter et al., 2014; Jiménez et al., 2015). *Novosphingobium* has been considered a potential laboratory contaminant in sequence-based microbiome analyses (Salter et al., 2014), but it has also been considered part of the “core” microbiome of human milk (Jiménez et al., 2015). Since it was one of the dominant OTUs in our study and it was more abundant in EBF in late lactation, it would suggest that *Novosphingobium* may be acquired in late lactation through retrograde flow (Ramsay et al., 2004). In relation to *Pseudomonas*, we observed the OTUs *Pseudomonas_MS_2* and *P. cedrina* in early EBF, *Pseudomonas_MS_2* was also more abundant in late EBF, and *Pseudomonas koreensis* and *P. fluorescens* were more abundant in early non-EBF. *Pseudomonas* is a genus that has been reported as dominant in other studies (Hunt et al., 2011; Jeurink et al., 2013; Boix-Amorós et al., 2016), and according to the latest systematic review in human milk, the microbiome *Pseudomonas* has been found in 50% of the studies analyzed with a relative abundance ranging from <1 to 17% (Zimmermann and Curtis, 2020).

In our study, we observed similarities in the shift of microbial phyla, genera, and species between early and late lactation. In EBF, the bacterial composition shifted from a higher abundance of the phyla Actinobacteria and Firmicutes in early lactation to a higher abundance of Bacteroidetes, Proteobacteria, and unknown bacteria in late lactation. This same shift also occurred in mothers who did not exclusively breastfeed. At the genera level, we observed a shift from *Corynebacterium, Staphylococcus*, and *Streptococcus* in early lactation to *Novosphingobium, Pseudomonas, Sphingobium*, and *Stenotrophomonas* in late lactation. These findings align with previous results that analyzed the impact of the stage of lactation and confirmed a shift from early to late stage (Gueimonde et al., 2007; Collado et al., 2008; Cabrera-Rubio et al., 2012; Khodayar-Pardo et al., 2014; Latuga et al., 2014; Chen et al., 2018; Gonzalez et al., 2021; Lopez Leyva et al., 2021b). In a study at the species level (Gonzalez et al., 2021), we also observed a shift from *Corynebacterium jeikeium, Lactobacillus gasseri, Staphylococcus hominis, Staphylococcus epidermidis, Streptococcus mitis, and Streptococcus parasanguinis* species in early lactation to *Sphingobium yanoikuyae, Pseudomonas putida*, and *Stenotrophomonas maltophilia* species in late lactation, but this previous study only analyzed the stage of lactation and did not consider the type of breastfeeding practice (Gonzalez et al., 2021). In the present study, we confirmed the presence of distinct species in those who breastfed at early and late lactation, indicating that the stage of lactation is an important modifier of the human milk microbiome. Our conclusion is that this general shift remains regardless of the breastfeeding practices (EBF or non-EBF).

### Association of exclusive breastfeeding with lactic acid bacteria and commensal bacteria

In EBF, more differentially abundant OTUs were observed at both stages of lactation, including lactic acid bacteria and commensal species with potential health benefits and specific functions. EBF had more abundance of lactic acid bacteria, including *Granulicatella elegans, Lactobacillales_MS_1, Lactobacillus gasseri, Streptococcus mitis*, and *Streptococcus parasanguinis*. The presence of these species is important due to the known benefits of LAB which include their ability to protect from harmful microorganisms and improve the nutrient acquisition of the host through their enzymatic functions (Vieco-Saiz et al., 2019). LAB can also counteract gastrointestinal infections, enhance lactose metabolism, minimize *Helicobacter pylori* infections, and strengthen immune responses (Ashraf and Shah, 2014). In fact, *Lactobacillus gasseri* has been recognized as a putative probiotic (Damaceno et al., 2017). Of note, both *Lactobacillus gasseri* and *Granulicatella elegans* were also present in the milk of mothers who did not exclusively breastfeed at early lactation. In contrast, when we compared EBF vs non-EBF at both stages of lactation, the addition of *agüitas* and/or complementary foods was associated with an overall lower abundance of LAB and commensal bacteria. Collectively, these results support the importance of WHO breastfeeding recommendations to continue exclusive breastfeeding for at least 6 months (World Health Organization [WHO], 2008), by showing a higher differential abundance of commensal and LAB associated with EBF.

### Environmentally sourced bacteria and breastfeeding practices

The presence of environmental bacteria in human milk remains a controversial area with regard to their source (Salter et al., 2014; Jiménez et al., 2015; Sakwinska et al., 2016; Douglas et al., 2020). A recent review that considered environmental sources of bacteria in human milk found that only 40% of human milk bacteria were isolated from human tissue, while the other 60% were first observed in association with the environment (Togo et al., 2019). In our study, we found environmental bacteria in mothers who exclusively breastfed, including *Brevundimonas_MS_1* and *Poaceae_MS_2* in early lactation and *Novosphingobium clariflavum_1, Comamonadaceae_MS_1, Pseudomonas cedrina_1, Pseudomonas_MS_2, Stenotrophomonas maltophilia_3, Pseudomonas_MS_1, Pseudomonas koreensis_1, Pseudomonas fluroescens_1, Cloacibacterium normanense*, and the OTU *Cloacibacterium_1* in late lactation, suggesting inoculation of human milk microbiome via either the entero-mammary pathway (Martín et al., 2004) or via retrograde flow (Ramsay et al., 2004). Alternatively, environmental bacteria could be introduced through water, or the herbs used for “*agüitas*”, the method of administration (spoon, bottle, or cup), or through the complementary foods provided to the infant. In our study, at early and late lactation, mothers who added *agüitas* and/or complementary foods also had a high abundance of environmental bacteria, including *Acinetobacter MS_1*, the OTUs *Corynebacterium_5, Corynebacterium_MS_2*, and *Corynebacterium tuberculostearicum_1* in early lactation and *Novosphingobium clariflavum_1, Comamonadaceae_MS_1, Pseudomonas fluorescens_1, Pseudomonas_MS_2, Pseudomonas koreensis_1, Stenotrophomona maltophilia_2*, and *Stenotrophomona maltophilia_ 6* in late lactation. Other studies have reported *Pseudomonas* and *Sphingomonas* as part of the “core” human milk microbiome (Hunt et al., 2011; Jiménez et al., 2015). The species *Novosphingobium clariflavum, Stenotrophomona maltophilia, Pseudomonas fluroescens*, and *Pseudomona koreensis* have been previously identified as species with extensive hydrocarbon degradation activity (Babalola and Ayangbenro, 2019; Gonzalez et al., 2021). Overall, there were fewer differentially abundant bacteria in the milk of mothers who did not exclusively breastfeed, but the associations and consequences of this on infant health will require further investigation.

### Strengths and limitations

This is the first study to characterize the DA of microbial species associated with EBF and to contrast it with shifts in the milk microbiome in mothers who did not exclusively breastfeed in the first 6 months of lactation. Our high-resolution analysis at the species level expands our understanding of the differences in milk microbiome composition, which may not be identifiable at the genera level. Finally, our milk collection methodology involved manual collection rather than the use of breast pumps, which has been shown to affect the milk microbiome analysis (Moossavi et al., 2019). Collectively, the aforementioned factors allowed us to characterize the human milk microbiome composition in a developing country and observe the impact of different breastfeeding practices at early and late lactation on the milk microbiome.

However, we recognize several limitations, including the cross-sectional nature of the study. Self-reported data, which were used to classify the mothers into the defined groups, may have introduced recall bias and could be a limitation. Moreover, we used the selected primers (27F/533R targeting the V1–V3 regions), which have high coverage for amplification of the genus *Cutibacterium* but not for species within the genus *Bifodibacterium* (Klindworth et al., 2012; Lopez Leyva et al., 2021a). Also, we are limited by the presence of several unknown or unidentified bacteria in the current libraries. Similarly, the lack of bacteriological studies of the water, soil, *agüitas*, and complementary foods limits the possibility of fully associating human milk microbiota with these factors.

## Conclusion

Understanding the impact of breastfeeding practices and the lactation stage on the milk microbiome is critical for advancing our comprehension of an optimal milk microbiome, and for supporting the current breastfeeding recommendations. Our findings highlight the importance of exclusive breastfeeding in promoting milk microbiota with more commensal bacteria during the first 6 months of lactation compared to non-exclusive breastfeeding. In contrast, non-exclusive breastfeeding before 6 months was associated with lower abundance of commensal and lactic acid bacteria. Collectively, these findings advance our understanding of the factors that affect the milk microbiome, which could provide preliminary evidence to further strengthen the recommendation by health practitioners for “exclusive breastfeeding on demand for 6 months” (World Health Organization [WHO], 2008) in order to establish a healthier infant microbiome.

Finally, this study addressed several informational gaps. These included the impact of feeding agüitas and complementary foods to the infant on the milk microbiome profile of rural indigenous mothers and the recognition of environmental bacteria as part of the milk microbiome. However, there is a need to close other gaps in the scientific literature that still exist, and future studies are required. This need includes uncovering associations of the human milk microbiome with sources of environmental bacteria, with functional properties and with early infant growth and development. Application and investment in more advanced technologies for human milk microbiome analyses will advance our current understanding of the human milk microbiome and its impact on infant health.

## Data availability statement

Publicly available datasets were analyzed in this study. This data can be found here: raw sequence data has been deposited at the European Genome-Phenome Archive (EGAD00001004160).

## Ethics statement

The studies involving human participants were reviewed and approved by the Institutional Review Board (IRB) of McGill University and the Center for Studies of Sensory Impairment, Aging and Metabolism (CeSSIAM). The patients/participants provided their written informed consent to participate in this study.

## Author contributions

KK, NS, EG, and LL: conceptualization, writing, reviewing, and editing of the manuscript. NS and KK: funding. All authors contributed to the article and approved the submitted version.

## Supplementary material

Supplementary File 1 Decontam ran on this dataset identified 1 OTU as putative contamination out of 1,505 OTUs. As expected, this OTU was not selected by DESeq2 as a candidate for differential abundance, due to low prevalence and high sparsity.

Supplementary File 2 A total of 503 OTUs were assembled and captured 77,827 sequence reads across all 64 human milk samples. These could be annotated as 287 OTUs, 134 genera, and 76 family or higher taxa, as well as 109 which could not be recognized as 99% similar (in both identity and coverage) to any known taxa and were labeled as unknown.

Supplementary File 3 Main factors were projected onto an unconstrained ordination diagram (NMDS), and each variable regression was independently tested by Monte Carlo permutation (envfit function from R package Vegan). The goodness of fit obtained is described in the table.

Supplementary File 4 The indices Chao 1, Shannon, and Observed used to estimate alpha diversity identified differences between early and late lactation for EBF and non-EBF mothers. Alpha-diversity indices were not significantly different in Shannon (*t*-test, *p* > 0.05) across the four groups. Differences in alpha diversity using Chao 1 were significant between early non-EBF and late EBF (FDR = 0.01) and between early EBF and late EBF (FDR = 0.04) groups, and using Observed, there were significant differences between early EBF and late EBF (FDR = 0.002), early non-EBF and late EBF (FDR = 0.005), and late EBF and late non-EBF (FDR = 0.08).

## Abbreviations

BLASTn: basic local alignment search tool
CAP: constrained analysis of principal coordinates
CeSSIAM: Center for Studies of Sensory Impairment, Aging, and Metabolism
DA: differential abundance
DNA: deoxyribonucleic acid
ebf: exclusive breastfeeding
FC: fold change
FDR: false discovery rate
LMIC: low- and middle-income countries
NCBI: National Center for Biotechnology Information
OTU: operational taxonomic unit
PCR: polymerase chain reaction
WHO: World Health Organization.
